# Recognizing Atypical Presentations of Alzheimer’s Disease: The Importance of CSF Biomarkers in Clinical Practice

**DOI:** 10.3390/diagnostics12123011

**Published:** 2022-12-01

**Authors:** George P. Paraskevas, Vasilios C. Constantinides, Fotini Boufidou, Ioanna Tsantzali, Efstratios-Stylianos Pyrgelis, Georgios Liakakis, Elisabeth Kapaki

**Affiliations:** 1 Neurochemistry and Biological Markers Unit, 1st Department of Neurology, School of Medicine, National and Kapodistrian University of Athens, Eginition Hospital, Vass. Sophias Ave. 74, 11528 Athens, Greece; 2 2nd Department of Neurology, School of Medicine, National and Kapodistrian University of Athens, “Attikon” University General Hospital, Rimini 1, 12462 Athens, Greece

**Keywords:** Alzheimer’s disease, tau, phospho-tau, amyloid-beta, cerebrospinal fluid biomarkers, atypical presentations

## Abstract

Besides the typical amnestic presentation, neuropathological studies indicate that Alzheimer’s disease (AD) may present with atypical clinical pictures. The relative frequencies of typical and atypical or mixed presentations within the entire spectrum of AD remain unclear, while some mixed or atypical presentations may have not received adequate attention for them to be included in diagnostic criteria. We investigated the spectrum of clinical presentations in patients with the AD CSF biomarker profile (high tau and phospho-tau, low Aβ42 levels), hospitalized in a tertiary academic center. Among 98 patients with the CSF AD profile, 46% of patients had the typical presentation of “hippocampal” amnestic dementia. Additionally, 23.5% and 15.3% fulfilled the criteria of mixed or atypical presentations, respectively, as described in the IWG-2 criteria. The remaining 15.3% had unusual presentations, including non-logopenic (semantic and non-fluent agrammatic) primary progressive aphasia, corticobasal syndrome, and Richardson syndrome, or could be diagnosed with normal pressure hydrocephalus. Despite selection bias (academic center), atypical clinical presentations of AD may be more common than previously thought. CSF biomarkers seem to be a useful tool for antemortem identification of such patients, which is likely to affect therapeutic decisions. Some of the unusual presentations described above should be incorporated in diagnostic criteria.

## 1. Introduction

Until relatively recently, the diagnosis of Alzheimer’s disease (AD) during life was mainly clinical and given to patients with an amnestic dementia syndrome according to criteria introduced 30 years ago [1]. With time, due to the results of pathological studies, it became evident that AD patients may present at pre-dementia stages [2] or with atypical pictures in the form of various syndromes, including primary progressive aphasia (PPA) [3], corticobasal syndrome (CBS) [4], posterior cortical atrophy (PCA) [5], and frontotemporal dementia (FTD)-like syndrome [6], or a mixture of other conditions, including cerebrovascular [7] and Lewy body pathology [8]. A major contribution for the in vivo recognition of these unusual presentations of AD came from the use of imaging [9] and, especially, cerebrospinal fluid (CSF) biomarkers [10,11], which can identify the biochemical “fingerprint” of AD during life. This expanded concept of AD [12] has been at least partly incorporated in recent diagnostic criteria [13,14,15]. Early and accurate diagnosis of unusual clinical presentations of AD, as well as exclusion of other primary [16] or secondary [17,18] causes of dementia, reduces uncertainty and may have significant implications in therapeutic decisions and predictions of prognosis.

However, the relative frequencies of typical and atypical or mixed presentations within the entire spectrum of AD remain unclear, while some mixed or atypical presentations may have not received adequate attention for them to be included in recent criteria. The aim of the present study was to explore the spectrum of clinical presentations in patients with the AD CSF biomarker profile who were hospitalized in a tertiary academic center.

## 2. Materials and Methods

### 2.1. Patients

Patients were consecutively hospitalized in a tertiary academic clinic between December 2013 and May 2016 and recorded without any selection in the Dementia and Movement Disorders Registry of the 1st Department of Neurology at Eginition Hospital. From this registry, we recruited patients showing the biochemical AD profile after determination of the 3 classical CSF biomarkers, namely amyloid peptide with 42 amino acids (Aβ_42_), total tau protein (τ_T_), and phospho-tau protein with phosphorylation of a threonine residue at position 181 (τ_P-181_), based on cut-off values of the Neurochemistry and Biological Marker Unit of our Department (Aβ_42_ ≤ 580, τ_T_ ≥ 376, and τ_P-181_ ≥ 62.5 pg/mL, respectively) [19]. It is generally suggested that abnormal Aβ_42_ and abnormal tau protein (either τ_T_ or τ_P-181_) are considered compatible with AD [13,15]. However, in the present study, for maximal specificity and according to more recent recommendations [20], the AD profile was pre-defined as abnormal values of all 3 biomarkers, i.e., decreased Aβ_42_ and increased τ_T_ and increased τ_P-181_ (thus, a patient with low Aβ_42_ and increased τ_T_ but normal τ_P-181_ was excluded). Finally, from a total of 243 consecutively hospitalized dementia patients, 98 showed the AD profile as defined above and were included in the study (only 1 additional patient had low Aβ_42_ and high τ_P-181_ but normal τ_T_ and was excluded). Following that, the various clinical, neuroimaging, and neuropsychological data (vide infra) were analyzed and the clinical picture of the patient was assigned to one of the subtypes of the International Working Group (IWG-2) criteria for Alzheimer’s disease [15]. Three neurologists with experience in neurodegenerative disorders (E.K., G.P.P., and V.C.C.) agreed on a classification, blinded to the CSF biomarker results. Additionally, all patients had at least a 3-year follow-up with no change in diagnosis. All patients and/or relatives gave written informed consent for inclusion in the study, which was performed according to the 1964 Declaration of Helsinki and with the approval of the Scientific and Ethics Committee of the Eginition Hospital.

### 2.2. Neuroimaging

All patients had a 3T MRI with visual assessment of medial temporal atrophy in T1 coronal sections [21]. When clinically indicated (suspicion of a Lewy body or other movement disorder), 131I-CIT dopamine transporter single photon emission computerized tomography (DaTscan) was performed.

### 2.3. Neuropsychological Testing

Following a complete physical and neurological examination, a battery of neuropsychological tests was performed in all subjects. This included tests of global cognitive function through use of the Clinical Dementia Rating (CDR) and Mini Mental State Examination (MMSE) [22,23], tests of memory using ‘The 5 words’ test [24], tests of frontal executive function using the Frontal Assessment Battery (FAB) [25], and tests of visuo-constructive abilities using the CLOX test [26]. According to the clinical presentation, when deemed necessary, additional tests for praxis (including the Goldenberg apraxia test [27,28]) and/or language (using The Boston Diagnostic Aphasia Examination [29]) were performed.

### 2.4. CSF Analysis

Lumbar puncture was performed at 10–11 AM, after overnight fasting, at the L4–L5 interspace according to recently proposed recommendations on standardized operating procedures (SOPs) for CSF biomarkers [30]. Four polypropylene tubes were used for CSF collection. The initial tube (2 mL) was used for routine cytology and biochemistry and the next tube (2 mL) for syphilis serology and any other determination needed by the clinical presentation. The last 2 tubes (5 mL each) were immediately centrifuged, aliquoted in 1 mL polypropylene tubes (3/4 of the tube filled, 750 μL each), and stored at −80 °C until analysis. Samples with more than 500 red blood cells/μL were discarded. Aliquots were thawed only once, just before analysis, which was performed within the first 6 months of storage in all subjects. The CSF levels of Aβ_42_, τ_P-181_, and τ_T_ were measured blindly in duplicate using a double-sandwich enzyme-linked immunosorbent assay (ELISA) as provided by commercially available kits (“Innotest hTau antigen”, “β-amyloid1–42”, and “phospho-tau181”, respectively, Fujirebio, Gent, Belgium), according to manufacturer’s instructions and with the use of a 4-parameter logistic curve.

### 2.5. Statistical Analysis

All variables were checked for normality and homogeneity of variances using the Shapiro–Wilk’s and Leven’s tests, respectively. The levels of CSF biomarkers did not follow the normal distribution and their variances were heterogeneous. Logarithmic transformation restored the above violations and permitted the use of parametric tests. The levels of the 3 CSF biomarkers were compared among groups by two-way analysis of covariance (2-way ANCOVA). One-way analysis of variance (1-way ANOVA), the Kruskal–Wallis test, and the χ2 test were also used as appropriate. The level of statistical significance was set at 0.05. For statistical analysis, the following software packages were used: Statistica version 8.0 (StatSoft Inc., Tulsa, OK, USA, 2008), Prism version 6.01 (GraphPad Software Inc., San Diego, CA, USA, 2012), and Medcalc^®^ version 12.5 (MedCalc Software, Ostend, Belgium).

## 3. Results

Almost half (46%) of the patients had the typical presentation of “hippocampal” amnestic dementia (Table 1).

**Figure 1:**
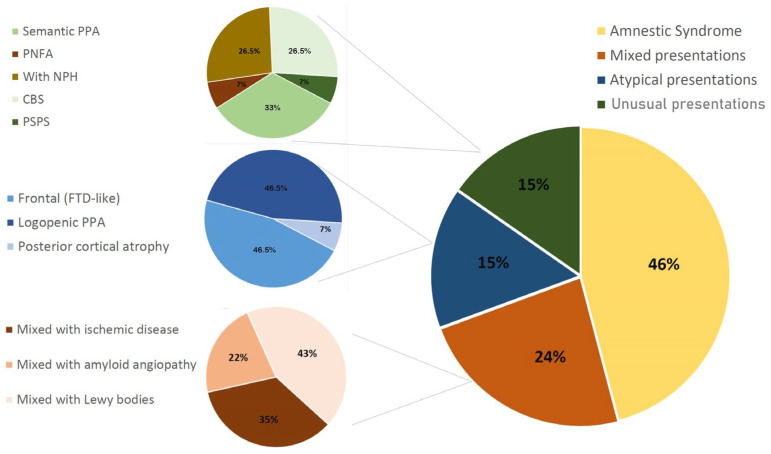
Pie chart of the clinical phenotypes of patients with an AD biochemical profile.

Additionally, 23.5% and 15.3% fulfilled the criteria of mixed or atypical presentations, respectively, as described in the IWG-2 criteria [15]. However, the remaining 15.3% had unusual presentations, including non-logopenic PPA fulfilling current criteria for semantic and non-fluent agrammatic (PNFA) PPA subtypes [31], corticobasal syndrome fulfilling criteria for probable corticobasal degeneration [32], Richardson syndrome fulfilling criteria for probable progressive supranuclear palsy (PSP) [33] or NPH diagnosis [34] (Figure 1).

Studied groups did not differ significantly in respect to sex, disease duration, and scores in neuropsychological tests (Table 2).

In the present cohort, 59% of patients had early (presenile) onset disease. Patients of the mixed group were older, had a higher age of symptom onset, and showed lower rates of early onset disease (<65 y) compared to the other groups.

**Figure 2:**
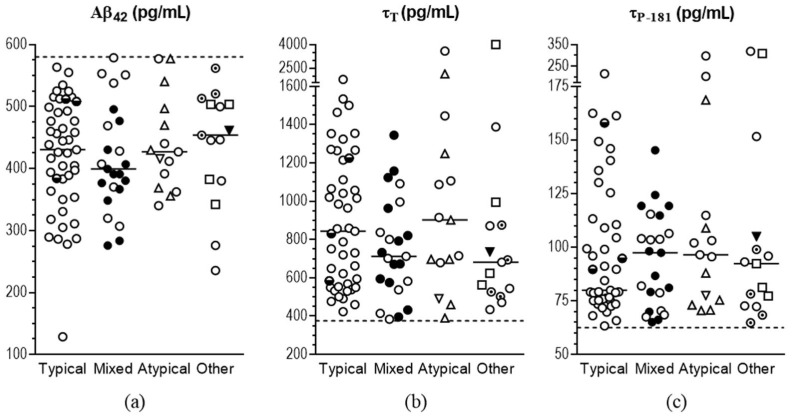
Scatterplot of CSF concentrations of Aβ_42_ (**a**), τ_T_ (**b**), and τ_P-181_ (**c**) in AD subgroups. Horizontal broken lines indicate the cut-off values of our laboratory. Horizontal bars indicate median values. For typical presentations, white circles indicate dementia stage and half-filled circles indicate MCI stage. For mixed presentations, black circles indicate mixed with VD and white circles indicate mixed with Lewy bodies. For atypical presentations, upward white triangles indicate frontal, downward white triangles indicate posterior, and white circles indicate logopenic presentations. For other unusual presentations, black triangles indicate PSP-like, white circles indicate non-logopenic PPA, dotted white circles indicate NPH, and white squares indicate CBS presentations.

Within the mixed group, no difference was noted between mixed with vascular disease and mixed with Lewy body disease regarding age, disease duration, MMSE, Aβ_42_, τ_P-181_, and τ_T_. After correction for multiple comparisons, only the logopenic subgroup showed a trend towards significantly higher median [quartiles] levels of τ_P-181_ [103 (97–203) pg/mL] compared to typical AD (*p* = 0.076). No significant difference was observed in any of the studied parameters between mixed with vascular and mixed with LB subgroups or among posterior, frontal, and logopenic subgroups (Figure 2).

## 4. Discussion

Atypical presentations of AD have been incorporated in most recent diagnostic criteria. However, recognizing atypical AD patients based on clinical, neuropsychological, or imaging data is difficult. CSF biomarkers represent a reliable tool of AD identification in vivo. Studies on CSF biomarkers in patients with diverse cognitive, behavioral, and/or movement manifestations aiming to identify atypical AD presentations are rare. These studies are important because they highlight the clinical diversity of AD and reiterate the importance of CSF biomarkers in identifying atypical AD phenotypes.

In the present study, we retrospectively reviewed the clinical presentations of 98 patients with a typical AD CSF biomarker profile. Roughly half of AD patients presented with the typical amnestic picture. This percentage is much lower compared to the 84% suggested by community-oriented studies [35] and is closer to the 54–63% observed in samples coming from research centers [36,37]. A selection bias seems to be responsible for this low incidence, since the study was conducted in a tertiary academic center where patients with atypical presentations are usually referred. Additionally, more than half of our patients had early-onset AD, an age group where atypical presentations are known to be overrepresented [38]. Atypical presentations may represent one third of young-onset AD patients [39]. Thus, the relative frequencies observed in the present study do not reflect those of the community, but of specialized research centers. This selection bias is not necessarily a drawback. In fact, it serves as a magnifying lens, allowing better recognition and study of non-amnestic AD presentations. Indeed, in addition to the “usual atypical” presentations (mixed, frontal, posterior, logopenic), we have identified a small percentage of “unusual atypical” presentations not yet recognized in diagnostic or research criteria for AD [13,14,15].

Non-logopenic PPA patients with AD have been described in neuropathological [3,40] and CSF biomarker studies, rendering biomarkers useful in identifying such patients in vivo [19,41,42,43]. Indeed, AD may be the underlying cause not only in more than half (~75–86%) of the patients with logopenic, but also in ~20% of the non-fluent agrammatic and ~16–35% of the semantic subtypes of PPA [19,44]. AD may be the underlying pathology in some of the patients fulfilling even the criteria for probable CBD [4], and, again, the classical CSF biomarkers may be helpful in identifying such patients, who may comprise 30–38% of corticobasal syndrome presentation [45,46]. Interestingly enough, we observed one patient (1% of our group) with an AD biomarker profile and typical Richardson syndrome presentation. This has been reported previously in up to 10% of PSP patients [46], though it is not clear if they represent atypical presentation of AD or mixed pathology.

The existence of mixed cases with NPH has been known for a long time. AD and/or cerebrovascular pathology may be present in a significant percentage of NPH patients, increasing with age [47]. CSF biomarkers may offer a tool for identification of AD coexistence [48] which, in turn, may affect therapeutic decisions since it may predict a worse neurosurgical outcome [49], although some positive effects in quality of life may still be observed [50]. However, such cases have not been included in the diagnostic criteria yet.

Results on biomarker levels among the various presentations of AD are scarce and conflicting. One study reported subtle differences in biomarker levels, with posterior cortical atrophy showing lower and the frontal variant showing higher levels of total tau and phospho-tau [36]. In another study, no significant differences were observed [37], and our results tend to agree with this latter study, with the exception of marginally higher τ_P-181_ levels in logopenic patients.

Our study lacks pathologic confirmation; however, this is an inherent limitation of the vast majority of biomarker studies. In order to reduce incorrect classification as much as possible, extensive investigation and a two-year follow-up were applied. Additionally, a strict definition of the “AD signature” was used (all three biomarkers abnormal) and our cut-off values were based on large healthy control and AD groups that had been previously studied [19].

## 5. Conclusions

Atypical clinical presentations of AD may be more common than previously thought and CSF biomarkers seem to be a useful tool for antemortem identification of most of these patients in everyday practice. It is not known whether currently available treatments for AD have the same efficacy in atypical as in typical amnestic presentations. However, accurate diagnosis is likely to affect therapeutic decisions both currently [51] and, especially, in the future, where new treatments targeting specific AD biochemical mechanisms are expected. In addition to assisting in the recognition of atypical and mixed AD presentations in vivo, the present study provides further insight into even rarer, unusual variants of AD, such as corticobasal syndrome. Non-logopenic language presentations, corticobasal syndrome presentations, and presentations mixed with NPH should be incorporated in newer versions of clinical or research criteria for AD. Rarely, mixed neurodegenerative pathologies may increase clinical diagnostic uncertainty, requiring postmortem pathological verification.

## Figures and Tables

**Table 1:** Clinical presentations of the patients with the AD CSF biomarker profile.

Clinical Presentation	Percentage	95% CI
Typical AD (“hippocampal” amnestic)	45.92%	33.49–61.44%
Mixed disease	23.47%	14.88–35.22%
Mixed with cerebrovascular disease	13.27%	7.06–22.68%
Mixed with ischemic disease	8.16%	3.52–16.09%
Mixed with amyloid angiopathy	5.10%	1.67–11.91%
Mixed with Lewy bodies	10.20%	4.89–18.77%
Atypical presentations	15.31%	8.57–25.25%
Frontal (FTD-like)	7.14%	2.87–14.72%
Logopenic PPA	7.14%	2.87–14.72%
Posterior cortical atrophy	1.02%	0.03–5.69%
Unusual presentations	15.31%	8.57–25.25%
Non-logopenic PPA	6.12%	2.25–13.33%
Semantic PPA	5.10%	1.67–11.91%
PNFA	1.02%	0.03–5.69%
With NPH	4.08%	1.11–10.45%
CBS	4.08%	1.11–10.45%
PSPS	1.02%	0.03–5.69%

CI: confidence interval, FTD: frontotemporal dementia, PPA: primary progressive aphasia, PNFA: progressive non-fluent aphasia of the agrammatic type, NPH: normal pressure hydrocephalus, CBS: corticobasal syndrome, PSS: progressive supranuclear palsy syndrome.

**Table 2:** Clinical and biochemical data of the studied groups.

	Typical	Mixed	Atypical	Unusual	*p* Value
*n* (%)	45 (45.92)	23 (23.47)	15 (15.31)	15 (15.31)	
f/m	26/19	12/11	9/6	6/9	NS *
Education (y) ^†^	12.3 ± 4.4	11.8 ± 4.9	11.1 ± 5.6	10.9 ± 4.5	NS ^§^
Age (y) ^†^	62.9 ± 11.6 ^a^	74.0 ± 5.8 ^b^	65.4 ± 5.9	70.5 ± 9.8	0.0007 ^§^
Age at onset ^†^	59.1 ± 12.1	70.1 ± 6.8 ^c^	60.7 ± 5.9	66.8 ± 9.1	0.0009 ^§^
Duration (y) ^†^	3.9 ± 3.6	3.8 ± 3.8	4.8 ± 4.2	4.0 ± 3.8	NS ^§^
Early onset (%)	33 (73.33)	6 (26.09) ^d^	11 (73.33)	8 (53.33)	0.0014 *
MMSE ^‡^	17 (12–20)	20 (10–24)	16 (7–22)	14 (5–21)	NS ^#^
FAB ^‡^	7 (3–13)	9 (4–14)	7 (4–12)	6 (2–13)	NS ^#^
5wIRt ^‡^	2.5 (0–4.75)	4.5 (3.25–5)	3.5 (0.75–5)	4 (0–5)	NS ^#^
5wDRt ^‡^	0.5 (0–2)	2 (0.25–5)	1 (0–5)	1 (0–5)	NS ^#^
CLOX1 ^‡^	4 (0–10)	5 (2–9)	3.5 (1.5–6)	3 (0.5–8)	NS ^#^
CLOX2 ^‡^	7 (0–12)	5 (4–10)	7 (3.75–11.25)	9 (2–13)	NS ^#^
VAMTA	3 (3–4) ^e^	3 (2–3)	2 (1–2)	2.5 (2–3)	0.001 ^#^
Aβ_42_ (pg/mL) **	430 (359–496) 421 ± 91.3	399 (367–477) 415 ± 86.2	427 (369–497) 440 ± 77.5	454 (380–503) 435 ± 94	NS ^††^
τ_T_ (pg/mL) **	844 (561–1220) 900 ± 354	713 (575–963) 754 ± 262	903 (679–1248) 1104 ± 819	682 (527–876) 927 ± 886	NS ^††^
τ_P-181_ (pg/mL) **	80 (76–112) 98.3 ± 33.4	97 (70–115) 94.2 ± 22.5	97 (75–115) 116 ± 62.3	92 (73–105) 119 ± 81.9	NS ^††^

f = females, m = males, y = years, NS = non-significant, MMSE = Mini Mental State Examination, FAB = Frontal Assessment Battery, 5wIRt = 5 words memory test–Immediate Recall total score, 5wDRt = 5 words memory test–Delayed Recall total score VAMTA = visual assessment of medial temporal atrophy. * χ2 test. † mean ± SD. ^a^ *p* = 0.077 vs. Other. ^b^ *p* = 0.0006 vs. Typical and 0.065 vs. Atypical. ^c^ *p* = 0.0009 vs. Typical and 0.042 vs. Atypical. ^d^ *p* = 0.0012 vs. Typical and 0.025 vs. Atypical. ^e^ *p* = 0.0003 vs. Atypical. § One-way ANOVA followed by Tukey’s post-hoc test. ‡ Median (25–75th percentile). # Kruskal–Wallis test followed by Dunn’s post-hoc test. ** Median (25–75th percentile) and mean ± SD. †† ANOVA after logarithmic transformation of original data or Kruskal–Wallis test.

## Data Availability

The data presented in this study are available upon reasonable request from the corresponding author. The data are not publicly available due to privacy restrictions.
